# Comprehensive CircRNA expression profile and selection of key CircRNAs during priming phase of rat liver regeneration

**DOI:** 10.1186/s12864-016-3476-6

**Published:** 2017-01-13

**Authors:** Lifei Li, Jianlin Guo, Yanhui Chen, Cuifang Chang, Cunshuan Xu

**Affiliations:** 1 College of Life Science, Henan Normal University, Xinxiang, 453007 Henan Province China; 2 State Key Laboratory Cultivation Base for Cell Differentiation Regulation and Henan Engineering Laboratory for Bioengineering and Drug Development, Xinxiang, 453007 Henan Province China

**Keywords:** High-throughput RNA sequencing technology, circRNA, Rat liver regeneration, miRNA, Host linear transcripts, Hepatocyte proliferation, Energy metabolism, Substance metabolism

## Abstract

**Background:**

Rat liver regeneration (LR) proceeds along a process of highly organized and ordered tissue growth in response to the loss or injury of liver tissue, during which many physiological processes may play important roles. The molecular mechanism of hepatocyte proliferation, energy metabolism and substance metabolism during rat LR had been elucidated. Further, the correlation of circular RNA (circRNA) abundance with proliferation has recently been clarified. However, the regulatory capacity of circRNA in rat LR remains a fascinating topic.

**Results:**

To investigate the regulatory mechanism of circRNA during priming phase of rat LR, high-throughput RNA sequencing technology was performed to unbiasedly profile the expression of circRNA during priming phase of rat LR. Gene ontology (GO) and Kyoto Encyclopedia of Genes and Genomes (KEGG) biological pathway analysis was conducted to predict the functions of differentially expressed circRNAs and their host linear transcripts. Co-expression networks of circRNA-miRNA were constructed based on the correlation analysis between the differentially expressed LR-related circRNAs and the condition of their miRNA binding sites. To excavate the key circRNAs in the early phase of rat LR, we comprehensively evaluated and integrated the relationship of expression level between the circRNAs and the linear transcripts as well as the distribution of miRNA binding sites in circRNA sequences.

**Conclusions:**

This paper is the first to employ the comprehensive circRNA expression profile and to investigate circRNA-miRNA interactions during priming phase of rat LR. Two thousand four hundred twelve circRNAs were detected, and 159 circRNAs deriving from 116 host linear transcripts differentially expressed (*p* < 0.05). Six significantly changed circRNAs during priming phase of rat LR were screened as key circle molecules, and then were validated by qRT-PCR. This study will lay the foundation for revealing the functional roles of circRNAs during rat LR and help solve the remaining clinical problems.

**Electronic supplementary material:**

The online version of this article (doi:10.1186/s12864-016-3476-6) contains supplementary material, which is available to authorized users.

## Background

LR is of great clinical significance in various liver-associated diseases [1]. As one of the vital organs in body, liver not only has the ability of detoxification, metabolism, biliation and immune defense, but has a high capacity to regenerate. Following 2/3 partial hepatectomy (PH), hepatocytes can be activated, and then enter cell cycle and proliferate to restore the original mass and function, which is called LR [2, 3]. LR is a complex process regulated by growth factors, cytokines and non-coding RNAs [4, 5].

CircRNAs, a novel type of non-coding RNAs, compose a class of RNA developing covalently closed loop structures without 5’–3’ polarities [6] but with widespread, abundant and tissue-specific expression across animals [7]. CircRNAs are generally formed by ‘backsplicing’ in which an upstream splice acceptor is joined to a downstream splice donor [8–12]. Although there isn’t consistent conclusion as to the function of circRNAs, a number of studies have revealed that circRNAs are classified into three types: circular exonic RNAs (ecircRNAs), circular intronic RNAs (ciRNAs) and exon-intron circRNAs (EiciRNAs) [6].

In recent years, the roles of circRNAs in the organism have been increasingly illuminated. CircRNAs can function as miRNA sponges, as templates for viroid and viral replication, as intermediates in RNA processing reactions, as regulators of transcription in cis, as snoRNAs (small nucleolar RNAs) [13], and so on. Different types of circRNAs may have distinct properties. CiRNAs and EiciRNAs might tend to be enriched in the nucleus and associate with the polymerase II elongation machinery to enhance expression of their corresponding parent genes [14, 15]. However, ecircRNAs have been identified to act as miRNA decoys to regulate the miRNA effects on their coding RNA targets by competing for miRNA binding, which seems like playing a regulation for competing endogenous RNA activities [7]. Hansen et al. [16] attested that circRNAs could act as miRNA sponges to regulate the expression of corresponding genes at the post-transcription level. For example, CDR1as (antisense to the cerebellar degeneration-related protein 1 transcript) with circularization into circRNA harboured 73 conserved binding sites for targeting miR-7, so it interacted with miR-7 and strongly suppressed miR-7 activity with resistant to miRNA-mediated target destabilization, resulting in increased levels of miR-7 targets [8]. Meanwhile, CDR1as was highly complementary to mature miR-671 and could be cleavaged by miR-671 [17]. Similarly, the transcript of sex-determining region Y (*SRY*) mainly in the form of circRNA in the adult testis [11] contained 18 conserved binding sites for miR-138, therefore it affected the target coding genes expression of miR-138. Nevertheless, some circRNAs had different conserved binding sites of different miRNAs, Yang [18] has verified that circ-FOXO3 could bind to eight miRNAs, effectively changing the translation of the target mRNA. Conclusively, circRNAs function as a sponge to bind a certain miRNA or multiple potential targeting miRNAs, resulting in changed gene expression.

A global reduction of circRNA abundance was reported in colorectal cancer [19], esophageal squamous cell carcinoma (ESCC) [20], gastric cancer [21], rectal cancer and ovarian cancer [19] compared to the peritumoral tissue, indicating that circRNAs might exert their important action in cell proliferation and apoptosis. Cell proliferation was the most important physiological activity during rat LR [22]. Consequently, an intriguing possibility was raised that alternative circRNA formation might constitute a novel regulatory layer in rat LR.

Salmena et al. hypothesized that these ceRNAs (competing endogenous RNAs), such as mRNAs, pseudogenes and long non-coding RNAs (lncRNAs), could communicate with each other through their ability to compete for miRNA binding. The communication generated wide-ranging cis and trans regulatory crosstalk across the transcriptome as a whole [23]. Recently enhancing evidences have indicated that circRNAs could function potent ceRNA molecules [6]. Much work has provided insights into the regulatory mechanism of non-coding RNA in rat LR including miRNA and lncRNA, little is known about the circRNA expression profile and the functions of circRNAs in rat LR. An in-depth study should be carried out to elucidate the potential role of circRNA, one of the ceRNAs, in rat LR. In this paper, high-throughput RNA sequencing technology was applied to detect the classification and expression level of circRNAs during priming phase of rat LR.it was Assumed that the circRNA function would be related to the known function of the host gene, we performed GO and KEGG biological pathway analysis to predict potential functions of circRNAs during priming phase of rat LR. LR-related circRNAs were further screened by analyzing the differentially expressed circRNAs, and then were validated by qRT-PCR.

## Results

### Expression pattern of circRNAs during priming phase of rat LR

**Fig. 1:**
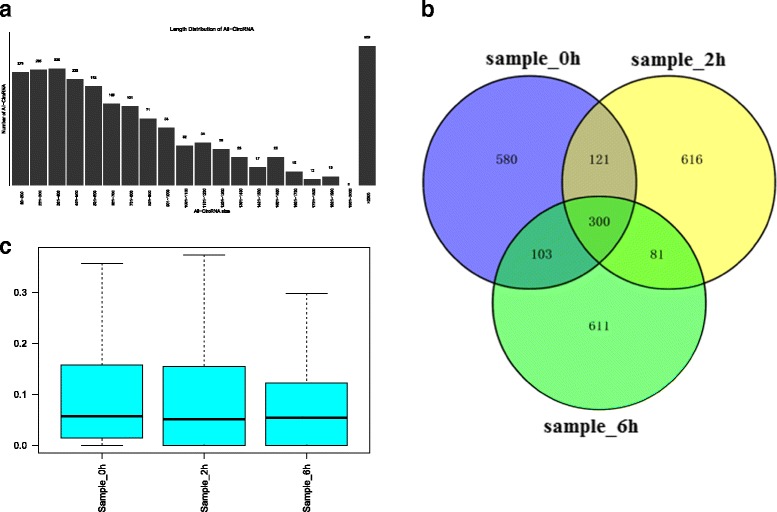
Expression pattern of circRNAs was detected by high-throughput RNA sequencing during priming phase of rat LR. **a** The length distribution of circRNAs. **b** Venn analysis of circRNAs detected at each time points. **c** Box plots of RPM value of circRNAs in three groups

High-throughput sequencing technology was used to detect the expression profile of circRNAs using the pipeline described by Memczak et al. [8], and the result showed that 2,412 circRNAs were identified in the regenerating rat livers at 0, 2 and 6 h after PH. The size of LR-related circRNA candidates ranged from under 100 nt to over 2000 nt, most of which was not very long in length. About 75% of circRNAs had the predicted spliced length of less than 2000 nt, wherein circRNAs with the length less than 500 nt were 45.56% and the length from 500 nt to 1000 nt were 21.43%. (Fig. 1a). Among the 2,412 circRNAs, 1104 circRNAs were detected at 0 h after PH, 1118 at 2 h, 1095 at 6 h, and 300 at all three time points (Fig. 1b), respectively. Expression abundance of circRNAs at three time-points after PH was measured based on RPM (mapped back-splicing junction reads per million mapped reads), and the result indicated that no abnormal expression was found in the three samples (Fig. 1c).

### Annotation of host linear transcripts and identification of differentially expressed circRNAs

**Fig. 2:**
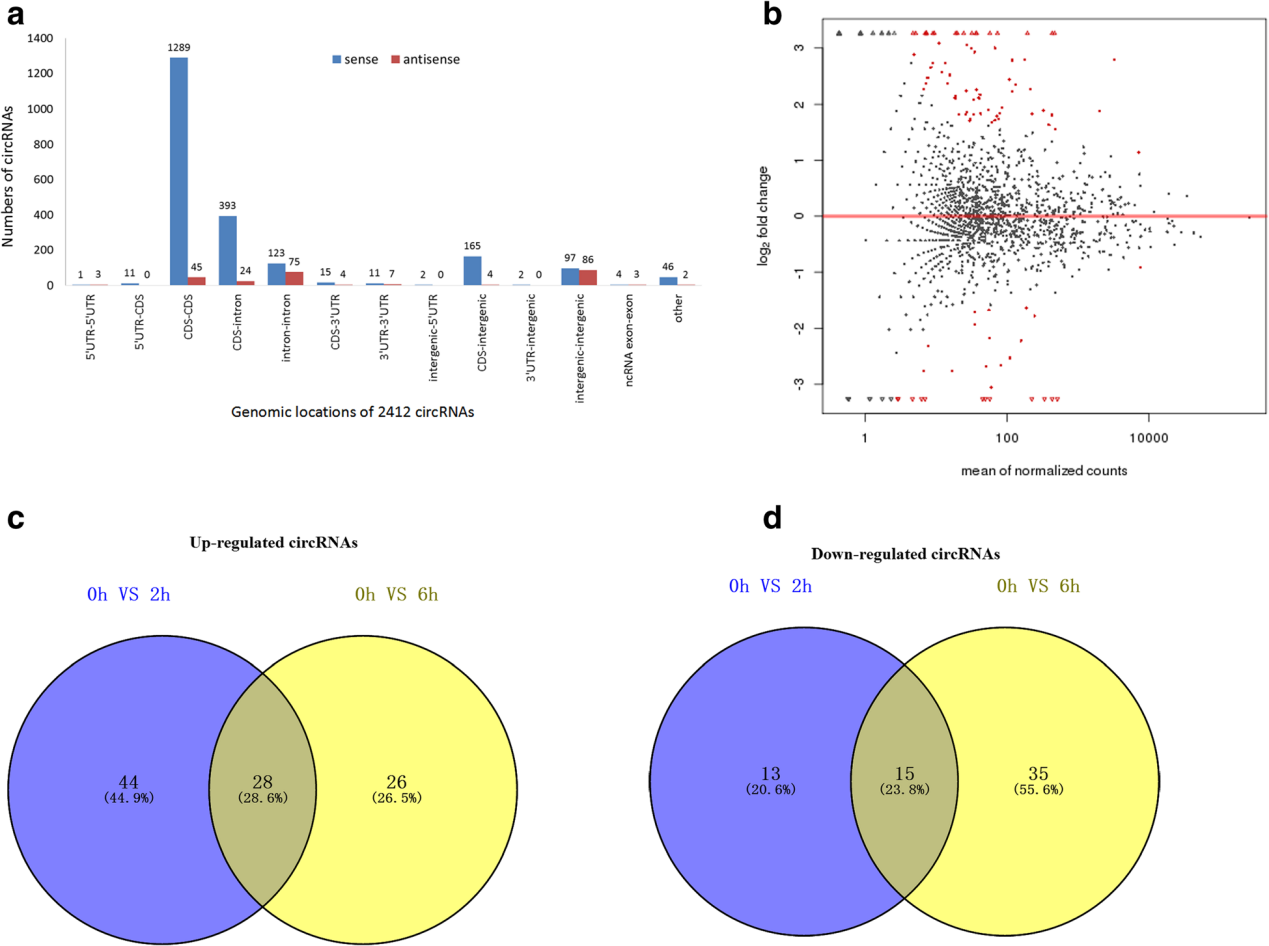
Annotation of Linear transcripts and identification of differentially expressed circRNAs. **a** The distribution of circular RNAs in the rat genome. **b** MA-plot of differentially expressed circRNAs at 2 h after PH compared with CG. **c** Differentially up-expressed circRNAs during priming phase of rat LR. **d** Differentially down-expressed circRNAs during priming phase of rat LR

Biogenesis of circRNAs via backsplicing was different from the canonical splicing of linear RNAs, but they all generally formed by alternative splicing of pre-mRNA. To provide a comprehensive landscape of the origination of circRNAs, linear transcripts of circRNAs from the corresponding genes were annotated and the circRNA distribution in the genome was also explored according to the location of the chromosome where the circRNA sequence was overlapped (Additional file 1). It proved that there were several distribution patterns of circRNAs in the gemome (Fig. 2a). One hundred fifty-nine differentially expressed circRNAs were selected during priming phase of rat LR. Among them, compared with control group (CG, 0 h), 100 differentially expressed circRNAs were selected by MA-plot with 72 up-regulated and 28 down-regulated at 2 h after PH (Fig. 2b and c), while 104 were selected with 54 up-regulated and 50 down-regulated at 6 h after PH (Additional file 2, Fig. 2c and d) which elucidated that many circRNAs were down-regulated at 6 h compared to 2 h after PH. Furthermore, at both 2 and 6 h compared with CG after PH, 45 circRNAs were differentially expressed with 28 circRNAs up-regulated and 15 circRNAs down-regulated, while only two circRNAs were regulated oppositely (Fig. 2c and d).

### The biomathematical predicted biological function of host linear transcripts

Under the assumption that circRNA function would be related to the known function of the host linear transcripts, differentially regulated linear transcripts were further analyzed by DAVID bioinformatics resources 6.7 (http://david.abcc.ncifcrf.gov). GO analysis were made on three different aspects namely biological process (BP), cellular component (CC) and molecular function (MF) for host linear transcripts. Prediction terms with *p*-value less than 0.05 were selected and ranked by *p*-value. According to the results, 463 and 340 GO terms were found up regulated (*p* < 0.05) at 2 and 6 h after PH. In contrast, 107 and 264 GO terms were found down regulated (*p* < 0.05). Top ten generally changed GO terms in all comparison groups classified by BP, CC, MF and ranked by *p*-value were listed. The consequence indicated that at 2 h after PH compared to GC, the most enriched and meaningful BP terms were superoxide metabolic process, cell aging, superoxide anion generation, and reactive oxygen species metabolic process. As for CC, the most enriched terms were mostly about focal adhesion, stress fiber and endoplasmic reticulum membrane. The most enriched MF terms were also closely related with the regulatory function of RNA polymerase II and enzyme activity involving in metabolic activity (Table 1). At 6 h after PH compared to GC, the most meaningful BP terms were cellular aldehyde metabolic process, long-chain fatty acid transport and lipid storage. As for CC, the most enriched terms were mostly about lipid particle, apical plasma membrane and basolateral plasma membrane. The most enriched MF terms were also closely related with enzyme activity involving in energy metabolism and substance metabolism, such as L-aspartate:2-oxoglutarate aminotransferase activity, hydroxymethylglutaryl-CoA synthase activity, glyceryl-ether monooxygenase activity and so on (Additional file 3). The results showed that during the early phase of rat LR, highly expressed host linear transcripts were mainly functionally involved in the response to stress, energy metabolism and the regulation of cell proliferation; all of these responses were helpful for the repair of the injured liver. Moreover, KEGG pathway analysis was made, and pathways (*p* < 0.05) were also selected and ranked by *p*-value. The top five were listed for differentially changed host linear transcripts at 2 and 6 h compared to GC. KEGG pathway analysis also showed that the top regulated ones were steroid hormone biosynthesis, inflammatory mediator regulation of TRP channels, and biosynthesis of amino acids at 2 h after PH compared to GC (Table 2). The top regulated ones at 6 h after PH were bile secretion, tyrosine metabolism, and fatty acid degradation (Additional file 4).

**Table 1:** GO annotations of host linear transcripts at 2 h after PH compared to GC

GO	*P*-value	GO terms
Biological process		
GO:0006801	0.000	superoxide metabolic process
GO:0007569	0.000	cell aging
GO:0042554	0.000	superoxide anion generation
GO:0072593	0.000	reactive oxygen species metabolic process
GO:0000902	2.38 × 10^−7^	cell morphogenesis
GO:2000573	4.12 × 10^−7^	positive regulation of DNA biosynthetic process
GO:0006536	3.63 × 10^−6^	glutamate metabolic process
GO:2000379	3.63 × 10^−6^	positive regulation of reactive oxygen species metabolic process
GO:0006744	2.80 × 10^−5^	ubiquinone biosynthetic process
GO:0043406	4.55 × 10^−5^	positive regulation of MAP kinase activity
Cellular component		
GO:0001725	0.000158	stress fiber
GO:0005925	0.000158	focal adhesion
GO:0005789	0.000333	endoplasmic reticulum membrane
GO:0016324	0.007339	apical plasma membrane
GO:0005769	0.028955	early endosome
GO:0005829	0.031131	cytosol
GO:0005887	0.05958	integral component of plasma membrane
GO:0005615	0.061762	extracellular space
GO:0070062	0.12631	extracellular vesicular exosome
GO:0043231	0.172681	intracellular membrane-bounded organelle
Molecular function		
GO:0000977	0.000	RNA polymerase II regulatory region sequence-specific DNA binding
GO:0001227	0.000	RNA polymerase II transcription regulatory region sequence-specific DNA binding transcription factor activity involved in negative regulation of transcription
GO:0004069	0.000	L-aspartate:2-oxoglutarate aminotransferase activity
GO:0004356	0.000	glutamate-ammonia ligase activity
GO:0016175	0.000	superoxide-generating NADPH oxidase activity
GO:0004707	4.63 × 10^−7^	MAP kinase activity
GO:0004497	1.80 × 10^−6^	monooxygenase activity
GO:0070330	4.04 × 10^−6^	aromatase activity
GO:0004838	4.55 × 10^−5^	L-tyrosine:2-oxoglutarate aminotransferase activity
GO:0020037	8.29 × 10^−5^	heme binding

**Table 2:** KEGG analysis of host linear transcripts at 2 h after PH compared to GC

Pathway	*P*-value	Pathway name
path:rno00140	0.000149	Steroid hormone biosynthesis
path:rno04750	0.006161	Inflammatory mediator regulation of TRP channels
path:rno01230	0.006938	Biosynthesis of amino acids
path:rno00591	0.01273	Linoleic acid metabolism
path:rno00590	0.031928	Arachidonic acid metabolism

### Construction of the circRNA-miRNA co-expression network

**Fig. 3:**
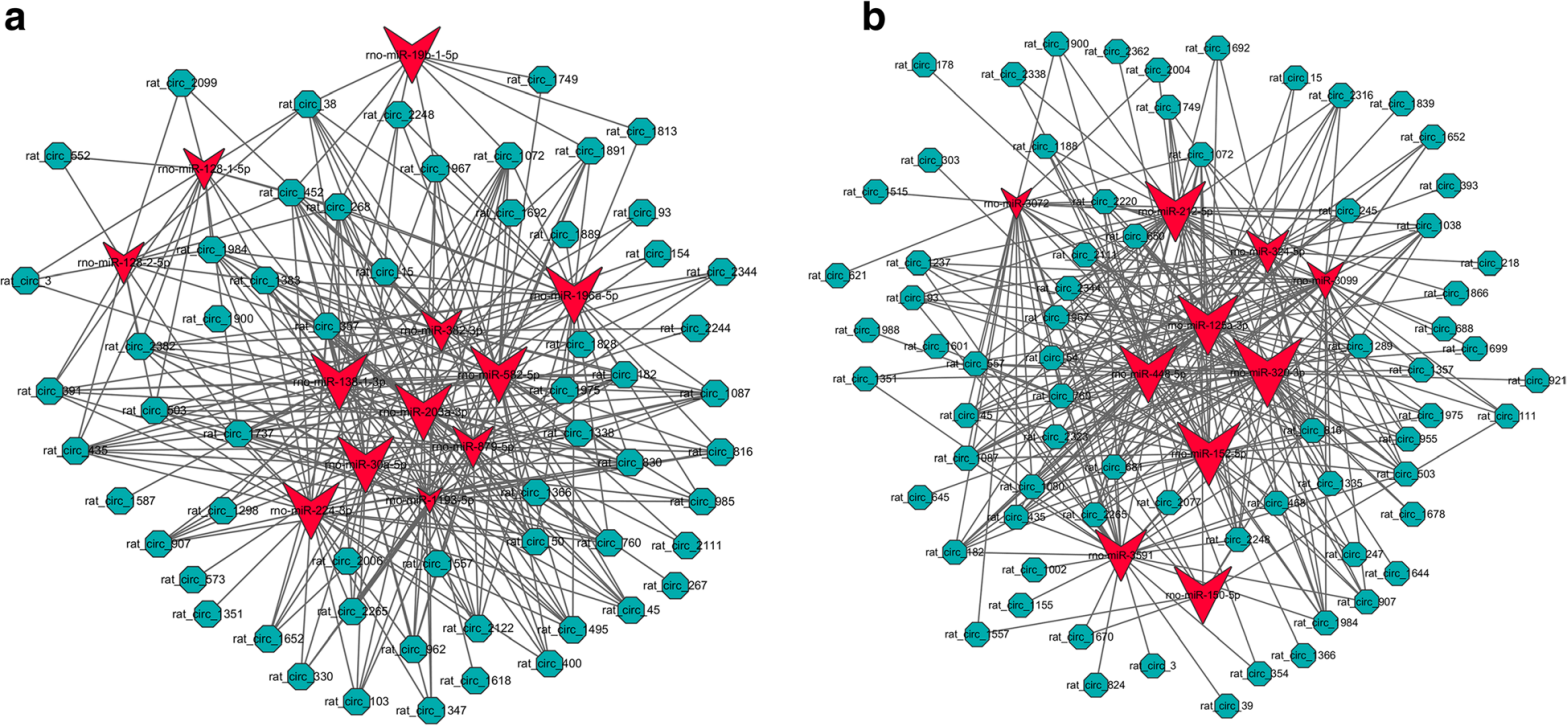
The circRNA-miRNA co-expression network. **a** Network of the circRNA-miRNA co-expression at 2 h compared with CG during rat LR. **b** Network of the circRNA-miRNA co-expression at 6 h compared with CG during rat LR. Circle nodes represent circRNAs and triangle nodes represent miRNAs. The size of circle and triangle represents *p*-value with larger size owing smaller *p*-value

In an attempt to reveal the co-expression pattern of circRNA-miRNA, the circRNA-miRNA co-expression networks were constructed based on the high-throughput RNA sequencing results. A constructed network map contained top 300 relationships between circRNAs and miRNAs ranked by *p*-value of the hypergeometric distribution (Fig. 3). In the network of the circRNA-miRNA co-expression at 2 h compared with CG, 12 miRNAs interacting with 59 circRNAs among the top 300 relationships were prognosed to have closer connections during rat LR (Fig. 3a). For example, miR-203a-3p was involved in liver regeneration, notably by inhibiting *SOCS3*, one known regulator of hepatic cell proliferation. Likewise, in the network of the circRNA-miRNA co-expression at 6 h compared with CG, ten miRNAs binding with 71 circRNAs had closer connections among which miR-150-5p had been verified to play a vital part during rat LR (Fig. 3b) [24].

### Screening of key circRNAs during priming phase of rat LR

After all circRNAs were screened during rat LR according to two methods, eight key circRNAs (circ1778, circ1366, circ15, circ3, circ2099, circ1338, circ2077, circ432) were selected according to method I (Table 3), and four circRNAs (circ2270, circ1678, circ137, circ1788) according to method II (Additional file 5). The key miRNAs related with rat LR were suppled to screen key circRNAs and were listed in Table 4. Based on the outcome of RNA high-throughput sequencing technology in rat liver, eight circRNAs selected out by method I were all up-regulated, and three of four circRNAs (circ2270, circ1678 and circ137) identified by method II were down-regulated during priming phase of rat LR.

**Table 3:**
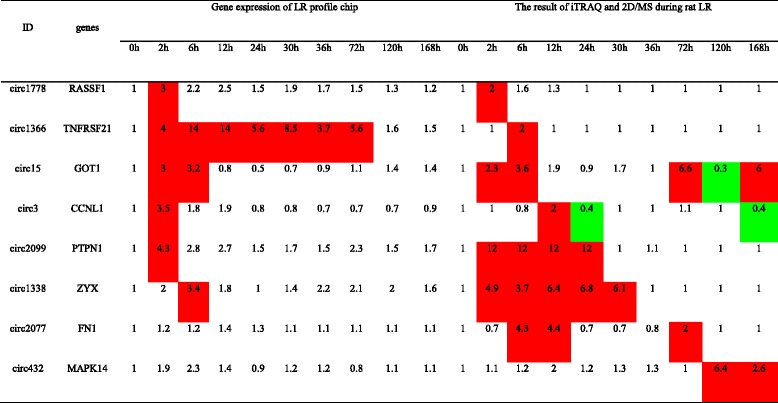
Eight circRNAs selected by method I

Red and green colors denote the expression level higher and lower than the control, respectively

**Table 4:** Key miRNAs during rat LR

miRNAs	Direct Target(s)	Variation tendency during rat LR	References	miRNAs	Direct Target(s)	Variation tendency during rat LR	References
miR-21	PELI1	up-regulated	[42]	miR-23b	SMAD3	down-regulated	[43]
	BTG2		[44]	miR-122	HO-1	up-regulated	[45–47]
	RHOB		[48]	miR-203	SOCS3	up-regulated	[49–53]
	PTEN		[54]	miR-382	PTEN	down-regulated	[35]
	FASLG		[55]		Akt	down-regulated	[35]
miR-221	P27	up-regulated	[56]	miR-16	-	down-regulated	[57]
	P57		[56]	miR-22	-	down-regulated	[57]
	ARNT		[58]	miR-23	-	down-regulated	[57]
miR-26a	CCND2	down-regulated	[59]	miR-24	-	down-regulated	[57]
	CCNE2		[59]	miR-29	-	down-regulated	[57]
miR-127	BCL6	down-regulated	[38, 60]	miR-30	-	down-regulated	[57]
	SETD8		[38, 60]	miR-31	-	down-regulated	[57]
miR-150	VEGF	down-regulated	[25]	miR-122a	-	down-regulated	[57]
miR-378	ODC1	down-regulated	[44]	miR-126	-	down-regulated	[57]
miR-181b	TIMP3	down-regulated	[61]	miR-145	-	down-regulated	[57]
miR-33	CDK6	down-regulated	[62]	miR-26b	-	up-regulated	[57]
	CCND1		[62]	miR-192	-	up-regulated	[57]
miR-34a	INHBB	up-regulated	[63]	miR-194	-	up-regulated	[57]
	MET		[63]				

### Verification of the expression changes of key circRNAs during rat LR by qRT-PCR

**Fig. 4:**
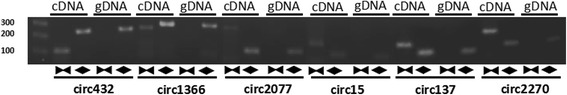
Outward-facing primers amplify circRNAs in cDNA but not genomic DNA (gDNA)

Twelve selected circRNAs were experimentally tested in 0 h PH group. Both outward-facing primers and opposite-directed primers were designed to detect the circular and linear form. Head-to-tail splicing existed only in circular form was assayed by quantitative polymerase chain reaction (qPCR) after reverse transcription, with outward-facing primers and Sanger sequencing. The result showed that the circular form was only amplified using the outward-facing primers and cDNA as templates, but it couldn’t be amplified using genomic DNA. PCR assays using both cDNA and genomic DNA could produce linear RNA using opposite-directed set (Fig. 4).

**Fig. 5:**
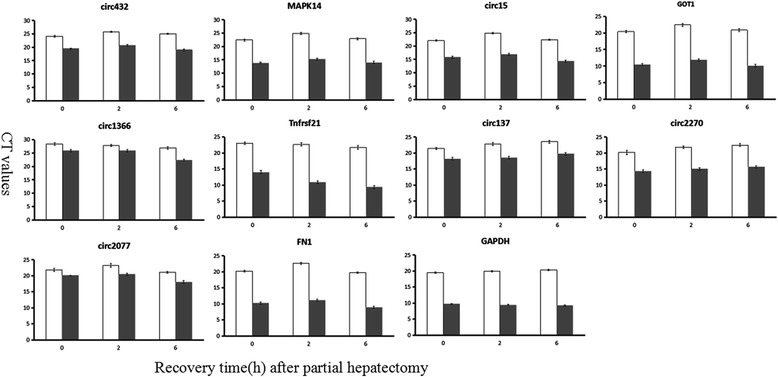
QRT-PCR analysis of differential decay of linear and circRNAs in the early phase of rat LR following the RNase R digestion. Expression levels were shown by CT values. (error bars indicate standard deviation)

**Fig. 6:**
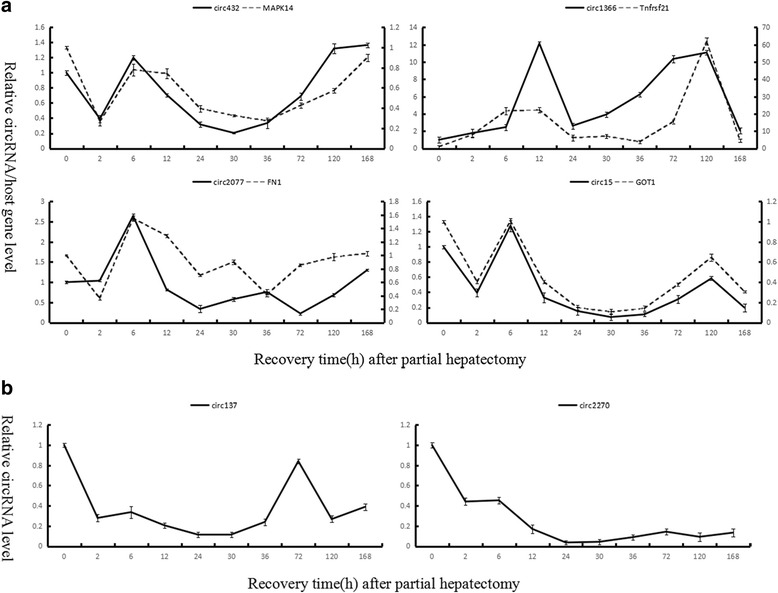
The expression changes of key circRNAs during rat LR by qRT-PCR. **a**The expression changes of four circRNAs selected by method I and their host linear transcripts during rat LR. **b** The expression changes of two circRNAs selected by method II during rat LR. Data represent the average value of at least three independent experiments

To confirm further the circular characteristics and explore the expression changes of 12 key circRNAs during priming phase of rat LR, six circRNAs and four host linear transcripts were randomly selected and amplified using outward-facing primers by qRT-PCR with RNase R digestion at 0, 2 and 6 h after PH. As expected, the circRNAs were more resistant to RNase R treatment than linear control RNAs (Fig. 5). Interestingly, the relative quantitative expression of the circRNAs and their host linear transcripts with or without RNase R digestion had the similar trends during priming phase of rat LR. To further verify the expression changes of six key circRNAs during rat LR, they were also amplified by qRT-PCR. These data demonstrated that circ432, circ2077, circ1366 and circ15 selected by method I significantly changed during rat LR, as well as the consequence showed positive correlation between four circRNAs and their corresponding origination genes, *MAPK14*, *FN1 (fibronectin 1)*, *TNFRSF21 (tumor necrosis factor receptor superfamily member 21)* and *GOT1* (*glutamic-oxaloacetic transaminase 1*), respectively (Fig. 6a). In addition, circ137 and circ2270 selected by method II were significantly down-regulated at ten time points during rat LR (Fig. 6b). Therein, four circRNAs (circ432, circ2077, circ1366 and circ15) selected by method I were up-regulated and two circRNAs (circ137 and circ2270) selected by method II were down-regulated during priming phase of rat LR, which had accordance with the high-throughput sequencing result even though the abundance relative expression values of the genes detected by above two methods were not all the same, demonstrating reliability of the RNA high-throughput sequencing technology.

## Discussion

Recently, the phenomenon of circRNA has gone from being perceived as a rare curiosity to having a central regulatory role in RNA metabolism [8, 11, 16, 25]. And the ever-growing discovery of circRNA with functional capacity showed that circRNA was a transcriptional product in various tissues and cell types of *archaea* [26], *human* [8, 10], *mouse* [8], *drosophila* [27] and *nematodes* [9] and in hundreds of cases constituted the dominant RNA isoform. Some circRNAs have been shown to affect microRNA regulation. Hansen et al. found that the circular transcripts of *SRY* had 18 miR-183 binding sites and that CDR1as had 73 miR-7 binding sites [16], so they proposed scientific hypothesis and demonstrated that circRNA could function as miRNA sponges to regulate the expression of target genes indirectly. Li et al. pointed out that cir-ITCH interacted with 3 molecules of miR-7, miR-17 and miR-214 to increase the level of *ITCH* and suppress the ESCC [20]. Wang et al. proved that HRCR (heart-related circRNA) could protect the heart from pathological hypertrophy and heart failure by targeting miR-223 [28]. Recently, Yang et al. demonstrated that circ-FOXO3 could function as a sponge to bind multiple potential targeting miRNAs, resulting in increased gene expression and inhibition of tumor growth and angiogenesis [18]. Thus, accumulating evidences suggested that circRNAs could function as miRNA sponges, and show distinct expression patterns coupled from their linear counterparts such as cir-ITCH and circ-FOXO3, or uncoupled from host gene expression such as CDR1as and HRCR.

To analyze the comprehensive circRNA expression profile by high-throughput RNA sequencing technology, 2,412 circRNAs were defined during priming phase of rat LR. One hundred fifty-nine differentially expressed circRNAs of them originated from 116 host linear transcripts. On one hand, 17 of 159 circRNAs were intergenic indicating that they did not have the corresponding linear transcripts. On the other hand, a gene could be spliced into one or more circRNAs [29]. Nineteen genes could produce more than one circRNAs during priming phase of rat LR, such as four circRNAs expressed from *Cpamd8*. The similar case had reported that *ANRIL* (antisense noncoding RNA in the INK4 Locus) encoded multiple, non-abundant linear and circular species and the expression of circular ANRIL isoforms (cANRIL) was correlated with INK4/ARF transcription and atherosclerotic vascular disease (ASVD) risk [30]. Furthermore, various circRNA isoforms from the same host linear transcripts expressed at different time points during priming phase of rat LR, implying the important function of temporal regulation of LR-related circRNAs.

To investigate the circRNA regulatory role in rat LR further, GO analysis was performed to annotate the biological functions of host linear transcripts during priming phase of rat LR, the result showed obviously that a significant amount of GO terms were related with the response to stress, energy metabolism and regulation of cell proliferation, which had been acknowledged as the important activities in the early stage of rat LR [1, 22, 31, 32]. The most enriched CC terms were about different kinds of plasma membranes. The plasma membranes were embedded with many proteins which could monitor and maintain the cell’s chemical climate and assist in the transfer of molecules across the membrane. The most enriched MF terms were closely related with diverse enzymes participating in energy metabolism and protein binding which was similar with circRNA function. Circ-FOXO3 interacted with the anti-senescent protein ID-1 and the transcription factor E2F1, as well as the anti-stress proteins FAK and HIF1a and retained them in the cytoplasm. As a result, these proteins could no longer exert their anti-senescent and anti-stress roles, resulting in increased cellular senescence [33]. The ciRNA, ci-ankrd52, largely accumulated to its sites of transcription, was associated with elongation Pol II machinery, and acted as a positive regulator of Pol II transcription [14]. In this way, circRNA might function as a scaffold for RNA binding proteins exerting its biological functions including regulating tanscription and so on. Coinciding well with the results of GO analysis, KEGG pathway analysis showed that linear transcripts were related with the signaling pathways closely linked to energy metabolism and substance metabolism including amino acid metabolism, fatty acid metabolism and bile secretion. All of them could provide a multitude of energy and materials to make preparation to trigger LR.

The amounting evidences have indicated that circRNAs might regulate the function of miRNAs acting as competing ceRNAs [8, 16, 20, 34]. In order to explore the role of the huge amount of circRNAs in rat LR, circRNA-miRNA co-expression networks were established to predict the relationships between circRNAs and miRNAs. Hitherto, there were no annotation of circRNAs in the circRNA-miRNA co-expression network, but some of the co-expressed miRNAs had already been proved functional crucially in rat LR. Exemplification showed that miR-150 regulated cell proliferation by targeting *VEGF* [25], besides miR-382 negatively regulated PTEN expression and increased Akt phosphorylation during LR [35]. From the co-expression network, the obvious conclusion was that miR-150 was related with nine circRNAs, and miR-382 was related with 22 circRNAs. Collectively, these results suggested that circRNAs might regulate rat LR by binding up to miRNAs that could regulate the associated gene expression.

Examining the roles of individual circRNAs will be the subject of future investigations. We screened the key circRNAs by two methods, and 12 key circRNAs during priming phase of rat LR were confirmed. Then, six of them were randomly selected to examine by qRT-PCR and sequenced correctly. Moreover, the expression of four circRNAs, circ432, circ2077, circ1366 and circ15 selected by method I was consistent with their linear transcripts *MAPK14*, *FN1*, *TNFRSF21* and *GOT1*, respectively, affirming that our methods were creditable. Simultaneously, the host genes of these four circRNAs have been interestingly substantiated to be vital for cell proliferation and energy metabolism. For example, MAPK14, one member of the four p38 MAPKs, played an important place in the cascades of cellular responses evoked by extracellular stimuli such as proinflammatory cytokines or physical stress. FN1 was a glycoprotein component of the extracellular matrix which functioned in embryogenesis, development, wound healing and metastasis. Blocking the adhesion function of FN1 by inhibiting its expression with miR-204-3p could promote the growth of hepatocellular carcinoma tumor endothelial cells [36]. Tumor necrosis factor (TNF) receptors were key factors in inflammation and immune regulation. Like other death receptors (DR), TNFRSF21, also known as DR6, could interact with TRADD, which had previously been shown to associate with TNFR1. Furthermore, ectopic expression of *TNFRSF21* in mammalian cells induced apoptosis and activation of both *NF-κB* and *JNK* [37]. GOT1 existed in cytoplasmic and mitochondrial forms and played a role in amino acid metabolism and the urea and tricarboxylic acid cycles. Hence, *MAPK14* was involved in a wide variety of cellular processes such as proliferation, differentiation, and development. *TNFRSF21* was strongly connected to tumorigenesis and apoptosis. *FN1* encoded structural protein involving in cell adhesion and migration processes. *GOT1* participated in energy and substance metabolism. Based on these investigation, A hypothesis was raised that circ432, circ2077, circ1366 and circ15 were novel and valuable parts in the process of hepatocyte proliferation, apoptosis, energy metabolism and substance metabolism during rat LR by altering the expression of their linear transcripts *MAPK14*, *FN1*, *TNFRSF21* and *GOT1*, respectively. In addition, circ137 and circ2270 selected by method II were both significantly down-regulated during rat LR. The outcome of biomathematical prediction manifested that circ137 and circ2270 could both couple with miR-127. Some experiments have demonstrated that miR-127 was capable of suppressing the hepatocyte proliferation, notably by inhibiting *BCL6* and *SETD8* [38]. Thus, circ137 and circ2270 might regulate the hepatocyte proliferation to control rat LR process by interacting with miR-127. Taken together, this paper provided the comprehensive landscape of circRNA expression profile during priming phase of rat LR and determined six representative circRNA candidates.

## Conclusions

In this work, 2,412 circRNAs were detected and 159 of them were preliminarily determined changing remarkably during priming phase of rat LR indicating their potential roles in rat LR. GO and KEGG biological pathway analysis indicated that host linear transcripts of circRNAs during the early phase of rat LR critically contributed to the process of energy metabolism, substance metabolism and hepatocyte proliferation. circ432, circ2077, circ1366 and circ15 might regulate the rat LR by controlling the expression level of *MAPK14*, *FN1*, *TNFRSF21* and *GOT1,* respectively, involved in networks critical for hepatocyte proliferation, energy metabolism and substance metabolism. It was also scientifically predicted that circ137 and circ2270 could regulate the hepatocyte proliferation by interacting with miR-127*.* So we suggest that expression of circRNAs sponging up miRNAs might play an important regulatory role as well. The findings will undoubtedly facilitate the future analyses of the regulatory mechanism of circRNA in rat LR, which may provide further insights into the comprehension of the regenerative process and have great promise to solve clinical problems.

## Methods

### Preparation of rat LR model

Adult Sprague–Dawley rats weighing 200 ~ 220 g were obtained from the Animal Center of Henan Normal University. They were kept in a controlled temperature room at 19 ~ 23 °C with a relative humidity of 50 ~ 70%, illumination of a 12 h period (8:00–20:00), and free access to water and standard rodent chow for two months. Experiments were performed among 18 rats with six animals per group: two PH groups and one normal (CG). The rats in PH groups were performed 2/3 PH according to the methods of Higgins [39]. The rats were anesthetized and sacrificed at 0, 2, and 6 h after surgery, and the right lobe of liver were instantly removed and stored at −80 °C in RNA Later. All experiments complied with the Animal Protection Law of China and animal Ethics.

### High-throughput RNA sequencing of circRNA related to rat LR

Liver tissues were harvested and pooled for total RNA extraction using TRIzol reagent. The quality of total RNA was tested by AGE (agarose gel electrophoresis) (100 V, 10 min). Mass, concentration, and purity of total RNA were measured by Spectrophotometer at 260 and 280 nm wavelengths. The samples with a ratio of OD260/280 of approximately 2.0 were used. CircRNAs were quantitatively analyzed by Shanghai OE Biotech (Shanghai, China). After removal ribosomal RNA and then building a library, a high-throughput RNA sequencing was performed. According to Memczak et al.’s methods [8], the clean reads were aligned to the reference genome by Bowtie2 (http://bowtie-bio.sourceforge.net/bowtie2/manual.shtml). For unmapped reads, the junctions were picked out using back-splice algorithm. Finally, circRNAs were verified with a software developed by OE which were considered as the reference sequence for further analysis. The expression level of circRNAs was measured by "Mapped back-splicing junction reads per million mapped reads" (RPM).

### Annotation of host linear transcripts and identification of differentially expressed circRNAs

Differentially expressed circRNAs were detected by the negative binomial distribution test based on the DESeq package. Two criteria were chosen: one was the foldchange of the same circRNA in two PH groups using 0 h group as control, and the other was *p*-value (*p* < 0.05) or FDR (false discovery rate) which was used to control filtration upon the statistics of alignment quality scores. Linear transcripts were annotated according to the location of the chromosome where the circRNA sequence was overlapped. Comparing the circRNA with genetic elements, the circRNA distribution in the genome could be explored.

### GO & KEGG pathway analysis of linear transcripts

DAVID (Database for Annotation, Visualization and Integrated Discovery) was used to analyze the potential functions of linear transcripts. Gene functions were classified into three subgroups namely BP, CC and MF. The top enriched GO terms among the two PH groups ranked by enrichment score were presented, and the top 10 enriched GO terms during priming phase of rat LR were ranked by the number of differentially expressed linear transcripts. KEGG pathway analysis was performed to determine the involvement of linear transcripts in different biological pathways.

### miRNA target prediction

Putative interactions between the miRNAs and circRNAs were evaluated using miRanda, investigating only perfect seed matching without gap of Wooble pairing (‘strict’ parameter). A hit between any expressed miRNA (including the new predicted miRNA) and a target circRNA was considered for a miRanda score of 140 or higher, corresponding to at least a perfect seed match.

### CircRNA-miRNA co-expression network analysis during priming phase of rat LR

Evidences have showed that circRNAs could bind with miRNAs and function as natural miRNA sponges to influence related miRNAs’ activities. CircRNA-miRNA co-expression network was built based on the prediction of miRNA binding sites and the correlations between circRNA and miRNA that was ranked by miRanda according to *p*-value of the hypergeometric distribution. The top 300 circRNA-miRNA were selected to generate a network map with cytoscape software (V. 3.2.1). Circle nodes represented circRNAs and triangle nodes represented miRNAs. The size of circle and triangle represented *p*-value with larger size stand for smaller *p*-value.

### The screening criteria of the key circRNAs during priming phase of rat LR

The correlation of circRNAs and origination genes was inconclusive. Some papers revealed the high correlation between circRNA abundance and expression levels of linear host genes across some cell lines [18], while others held the different view [19] that circRNA expression was often uncoupled with host gene expression [40, 41]. As a consequence, two methods were applied to screen the key circRNAs during rat LR.

Method I: Firstly, circRNAs were selected with foldchange ≥ 2 or foldchange ≤ 0.5 in expression levels and *p* < 0.05 in PH groups compared with CG. Then, the mRNA level of the host genes at ten time points of 0, 2, 6, 12, 24, 30, 36, 72, 120, and 168 h during rat LR was profiled using Rat Genome 230 2.0 array. Meanwhile the level of proteins coded by the host genes at ten time-points was investigated by two-dimensional differential gel electrophoresis (2D) and mass spectrometry (MS). The genes with mRNA expression change over 3 times and corresponding proteins change over twice were defined as significantly expression genes. If circRNAs and their host genes could both comply with the above standards, circRNAs were regarded as key circRNAs in rat LR.

Method II: This criterion was provided as following. Firstly circRNAs were selected that both conformed to *p* < 0.05 and foldchange ≥ 2 or foldchange ≤ 0.5 in PH groups compared with CG. Secondly, ecircRNAs were prioritized. Thirdly, the circRNAs with high expression levels may have the much possibility of developing more important functions. Last, the distribution of miRNA binding sites in circRNA sequence was predicted by miRanda (http://www.microrna.org/) software. The key miRNAs in rat LR confirmed in other papers such as miR-221 and miR-34a had the priority to be picked out, then circRNAs harbouring more than one above-mentioned miRNA binding sites were sorted out.

### RNase R digestion

Rat LR DNase-treated total RNA (6 mg) was incubated 1 h at 37 °C with zero units (mock treatment) or 20 units of RNase R (Epicentre Biotechnologies). RNA was subsequently purified by phenol-chloroform extraction, retro-transcribed and used in qRT-PCR.

### The expression level of circRNAs verification by qRT-PCR assay

To validate the reliability of high-throughput RNA sequencing and explore the expression trend of circRNAs during rat LR, the expression level of circRNAs was examined by qRT-PCR.

Reference to Memczak’s method [8], two sets of primers for each circRNAs were designed using the Primer Express software version 5.0 (Table 5): a outward-facing set which was expected to amplify only the circRNAs, and an opposite-directed set to amplify the linear forms.

**Table 5:** The primers sequence used in this study

id	primer1	primer2	product length(bp)
circ432_outward-facing	AGGTCCCATCTCCAGCACTATT	CAAATACACCTGTGATCTGAGCA	107
circ432_opposite-directed	TGCTTCCTCACTCCAGCTACG	CGGTTCTCCCTTTGTTCGG	239
circ2077_outward-facing	GCTGAGCAAGGAGGAAGAAATC	CACAGCCCAAAGTGTGAACATC	263
circ2077_opposite-directed	ATCACGCTGCTGGGACTTCCT	TGATACGCCCATTGCCTTCG	108
circ1366_outward-facing	AAGAACTGACGGATACTGGC	ACTCAGGCTCTTCTACCACATA	239
circ1366_opposite-directed	CATCACAGCCCAACCAGAACA	GTCAGTCAAGGCAGCACAAGG	271
circ15_outward-facing	AGTGAACAGCAACGGTGAA	AGGCAAGGCCAATAAGAAC	158
circ15_opposite-directed	TGTGCAGTCTTTGGGAGGGA	ACGGGCGTGTTCTTGTTGTC	93
circ137_outward-facing	TGATGCTGTCGTGGCTTCTGA	TGGTCCTGTGTCCACTAAATTCC	151
circ137_opposite-directed	GACAGCAAGGAGGTCAAAACC	GGCACCCGTAGGTCATTCATA	101
circ2270_outward-facing	AGAGCGTCCAACCTGAATTGAA	CACCAACCCAACGAGCATCAT	226
circ2270_opposite-directed	CCGAATAACTCCTAATGATGCTC	TTCCTTTCCTCCTGTGACCTCT	138

Then total RNA was extracted, digested using RNase R and purified, cDNA was synthesized using the AMV reverse transcription kit (Promega, USA). Outward-facing primers were designed to amplify the fragment across the junction from cDNA, then the fragment was sequenced by Sangon Biological Engineering Company (Shanghai China). QRT-PCR was performed using Q-SYBR green Supermix (BioRad), and PCR-specific amplification was conducted in Rotor-Gene 3000 (Corbett Robotics, Australia). The expression of circRNAs was defined based on the threshold cycle (Ct), and relative expression levels were calculated via the 2^-ΔΔCt^ method. GAPDH was served as internal standard control, and all reactions were performed in triplicate.

## Additional files

Additional file 1: The results of annotating circRNA host linear transcripts and analysing differentially expressed circRNAs at 2 h and 6 h after PH compared with CG. (XLSX 71 kb)
Additional file 2: MA-plot of differentially expressed circRNAs at 6 h after PH compared with CG. (PNG 14 kb)
Additional file 3: GO annotations of host linear transcripts at 6 h after PH compared to GC. (DOCX 17 kb)
Additional file 4: KEGG analysis of host linear transcripts at 6 h after PH compared to GC. (DOCX 13 kb)
Additional file 5: 8 circRNAs selected by method Method II. (XLSX 10 kb)

## Abbreviations

2D: Two-dimensional differential gel electrophoresis
AGE: Agarose gel electrophoresis
ANRIL: Antisense noncoding RNA in the INK4 Locus
ASVD: Atherosclerotic vascular disease
BP: Biological process
cANRIL: Circular ANRIL
CC: Cellular component
CDR1as: Antisense to the cerebellar degeneration-related protein 1 transcript
ceRNAs: Competing endogenous RNAs
CG: Control group
DAVID: Database for annotation, visualization and integrated discovery
DR: Death receptors
ESCC: Esophageal squamous cell carcinoma
FDR: False discovery rate
FN1: Fibronectin 1
GO: Gene ontology
GOT: Glutamic-oxaloacetic transaminase
HRCR: Heart-related circRNA
KEGG: Kyoto Encyclopedia of Genes and Genomes
lncRNA: Long non-coding RNAs
LR: Liver regeneration
MF: Molecular function
MS: Mass spectrometry
PH: Partial hepatectomy
RPM: Mapped back-splicing junction reads per million mapped reads
SRY: Sex-determining region Y
TNF: Tumor necrosis factor
TNFRSF21: Tumor necrosis factor receptor superfamily member 21
