# Supplementary figures and images for: Comprehensive CircRNA expression profile and selection of key CircRNAs during priming phase of rat liver regeneration
Source: BMC Genomics. 2017 Jan 13;18:80. doi: 10.1186/s12864-016-3476-6 (PMC5237265; doi:10.1186/s12864-016-3476-6)

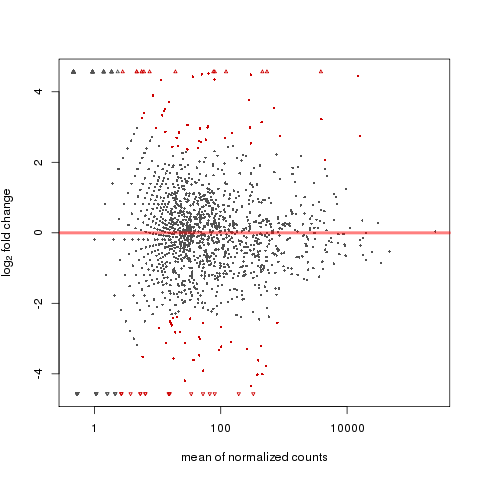

Supplement: Additional file 2: — MA-plot of differentially expressed circRNAs at 6 h after PH compared with CG. (PNG 14 kb) [file 12864_2016_3476_MOESM2_ESM.png]
