# Supplementary material for: Comprehensive CircRNA expression profile and selection of key CircRNAs during priming phase of rat liver regeneration
Source: BMC Genomics. 2017 Jan 13;18:80. doi: 10.1186/s12864-016-3476-6 (PMC5237265; doi:10.1186/s12864-016-3476-6)
Supplement: Additional file 3: — GO annotations of host linear transcripts at 6 h after PH compared to GC. (DOCX 17 kb) [file 12864_2016_3476_MOESM3_ESM.docx]

**Additional file 3: GO** **annotations of host linear transcripts at 6h after PH compared to GC.**

| GO | *P*-value | GO terms |
| --- | --- | --- |
| **Biological process** | | |
| GO:0006081 | 0.000 | cellular aldehyde metabolic process |
| GO:0015909 | 8.46×10^-6^ | long-chain fatty acid transport |
| GO:0019915 | 8.46×10^-6^ | lipid storage |
| GO:0006643 | 3.54×10^-5^ | membrane lipid metabolic process |
| GO:0046485 | 3.54×10^-5^ | ether lipid metabolic process |
| GO:0046686 | 0.000123713 | response to cadmium ion |
| GO:0014070 | 0.000195959 | response to organic cyclic compound |
| GO:0006536 | 0.000238971 | glutamate metabolic process |
| GO:0018149 | 0.000238971 | peptide cross-linking |
| GO:0051384 | 0.001202347 | response to glucocorticoid |
| **Cellular component** | | |
| GO:0005811 | 6.27×10^-5^ | lipid particle |
| GO:0016324 | 6.71×10^-5^ | apical plasma membrane |
| GO:0016323 | 0.000259 | basolateral plasma membrane |
| GO:0005829 | 0.001924 | cytosol |
| GO:0031012 | 0.005384 | extracellular matrix |
| GO:0005578 | 0.018021 | proteinaceous extracellular matrix |
| GO:0016021 | 0.018402 | integral component of membrane |
| GO:0005604 | 0.026194 | basement membrane |
| GO:0005764 | 0.027465 | lysosome |
| GO:0031410 | 0.027465 | cytoplasmic vesicle |
| **Molecular function** | | |
| GO:0004069 | 0.000 | L-aspartate:2-oxoglutarate aminotransferase activity |
| GO:0004421 | 0.000 | hydroxymethylglutaryl-CoA synthase activity |
| GO:0051287 | 1.84×10^-5^ | NAD binding |
| GO:0050479 | 3.54×10^-5^ | glyceryl-ether monooxygenase activity |
| GO:0005506 | 5.13×10^-5^ | iron ion binding |
| GO:0004838 | 0.000104 | L-tyrosine:2-oxoglutarate aminotransferase activity |
| GO:0005201 | 0.000239 | extracellular matrix structural constituent |
| GO:0004497 | 0.007671 | monooxygenase activity |
| GO:0070330 | 0.021103 | aromatase activity |
| GO:0020037 | 0.021311 | heme binding |

**Additionla file 3: KEGG analysis of host linear transcripts at 6h after PH compared to GC.**

| pathway | *P*-value | Pathway name |
| --- | --- | --- |
| path:rno04976 | 0.003238 | Bile secretion |
| path:rno00350 | 0.003238 | Tyrosine metabolism |
| path:rno00071 | 0.003238 | Fatty acid degradation |
| path:rno04726 | 0.027736 | Serotonergic synapse |
| path:rno00140 | 0.063602 | Steroid hormone biosynthesis |
