# Supplementary material for: Comprehensive CircRNA expression profile and selection of key CircRNAs during priming phase of rat liver regeneration
Source: BMC Genomics. 2017 Jan 13;18:80. doi: 10.1186/s12864-016-3476-6 (PMC5237265; doi:10.1186/s12864-016-3476-6)
Supplement: Additional file 4: — KEGG analysis of host linear transcripts at 6 h after PH compared to GC. (DOCX 13 kb) [file 12864_2016_3476_MOESM4_ESM.docx]

**Additional file 4: KEGG analysis of host linear transcripts at 6h after PH compared to GC.**

| pathway | *P*-value | Pathway name |
| --- | --- | --- |
| path:rno04976 | 0.003238 | Bile secretion |
| path:rno00350 | 0.003238 | Tyrosine metabolism |
| path:rno00071 | 0.003238 | Fatty acid degradation |
| path:rno04726 | 0.027736 | Serotonergic synapse |
| path:rno00140 | 0.063602 | Steroid hormone biosynthesis |
